# Expression and regulation of immune-modulatory enzyme indoleamine 2,3-dioxygenase (IDO) by human airway epithelial cells and its effect on T cell activation

**DOI:** 10.18632/oncotarget.11586

**Published:** 2016-08-24

**Authors:** Wejdan A. Aldajani, Fabián Salazar, Herb F. Sewell, Alan Knox, Amir M. Ghaemmaghami

**Affiliations:** ^1^ Division of Immunology, School of Life Sciences, Faculty of Medicine and Health Sciences, University of Nottingham, Nottingham, UK; ^2^ Division of Respiratory Medicine, School of Medicine, Faculty of Medicine and Health Sciences, University of Nottingham, Nottingham, UK

**Keywords:** epithelial cells, indoleamine 2,3-dioxygenase, T cell proliferation, allergen, Immunology and Microbiology Section, Immune response, Immunity

## Abstract

Indoleamine 2,3-dioxygenase (IDO) catalyzes the degradation of tryptophan, which plays a critical role in immune suppression through regulating the production of a series of metabolites that are generally referred to as kynurenines. It has become increasingly clear that epithelial cells (ECs) play an active role in maintaining lung homeostasis by modulating the function of immune cells via producing cytokines, chemokines, and anti-microbial mediators. In this study we assessed the regulation of IDO activity and expression in human primary ECs and EC lines under steady state conditions and in response to bacterial and allergenic stimuli. We also investigated the potential immune modulatory functions of IDO expression in human airway ECs. Our data clearly show that airway ECs produce IDO, which is down-regulated in response to allergens and TLR ligands while up-regulated in response to IFN-γ. Using gene silencing, we further demonstrate that IDO plays a key role in the EC-mediated suppression of antigen-specific and polyclonal proliferation of T cells. Interestingly, our data also show that ECs lose their inhibitory effect on T cell activation in response to different TLR agonists mimicking bacterial or viral infections. In conclusion, our work provides an understanding of how IDO is regulated in ECs as well as demonstrates that “resting” ECs can suppress T cell activation in an IDO dependent manner. These data provide new insight into how ECs, through the production of IDO, can influence downstream innate and adaptive responses as part of their function in maintaining immune homeostasis in the airways.

## INTRODUCTION

Epithelial cells (ECs) are the first line of defense against airborne pathogens and allergens. ECs modulate immune responses by regulating the expression of different pattern recognition receptors and *via* their own ability to produce a plethora of cytokines and chemokines. Furthermore, it is well established that the cross-talk between ECs and dendritic cells (DCs) is very important in orchestrating immune responses to airborne antigens. In this context, ECs have been shown to directly and indirectly modulate T cell responses [1, 2]. In particular, airway ECs can influence T cell activation and differentiation by increasing the recruitment, maturation, and activation of DCs through the secretion of diverse chemokines [3–5] and cytokines [6, 7]. For example, murine colonic [8] and lung [9, 10] ECs are able to inhibit antigen presenting cell-induced T cell proliferation. This effect appears to be cell-cell contact-dependent [8–10], and was found to be attenuated by pre-treatment of ECs with IL-4 [10] or after viral infection [9]. In addition, it has been suggested that direct contact between ECs and DCs is essential to inhibit *in vitro* T cell responses against allergens [11]. However, despite some evidence suggesting a role for TGF-β in decreasing T cell proliferation to some extent, the exact mechanism underlying such EC-mediated suppression of T cell responses has remained elusive [9].

Tryptophan (TRP) is an essential amino acid for the synthesis of proteins and neurotransmitters as well as for cell growth and function [12]. In mammals, the primary route of TRP degradation into kynurenines (KYNs) is controlled by extra-hepatic indoleamine 2,3- dioxygenase (IDO) and hepatic tryptophan 2,3-dioxygenase. There are two IDO isoforms, IDO1 and IDO2 [13–15], and these isozymes exhibit different expression patterns and molecular regulation [12, 15, 16]. However, the function of IDO1 (herein referred to as IDO) has been more extensively analyzed and was shown to have diverse immune-regulatory properties [17, 18]. TRP depletion as well as TRP-derived metabolites can impact T cell activation by inducing apoptosis, activating the stress-response kinase GCN2, or promoting tolerance through activation of the aryl-hydrocarbon receptor [19, 20]. DCs express high levels of IDO in response to different stimuli, including cytokines such as type-I and type-II IFNs, co-stimulatory molecules, and TLRs [21]. IDO is highly expressed in the immune cells; however, non-immune cells, including ECs, have also been shown to express functional IDO [22]. Previous work has shown an increase in IDO activity and expression (at the mRNA level) in human cervical ECs (HeLa cells) after stimulation with IFN-γ [23, 24]. This effect was further enhanced in the presence of IL-1 or TNF-α, but not in response to LPS stimulation. Furthermore, it was demonstrated that diverse epithelial carcinoma cell lines [25–27] and primary ECs [28, 29] express IDO after IFN-γ treatment. In addition, functional IDO expression has been reported to be high in the lung [30]. More recently, it was demonstrated that *Aspergillus fumigatus* spores induced the up-regulation of IDO in corneal ECs, suggesting the involvement of IDO from ECs in the immune responses against fungal infections [31].

The aim of this study was to investigate the regulation of IDO expression and activity in airway cancerous and non-cancerous ECs in response to TLR agonists and allergen extracts; and to investigate the potential role of EC-derived IDO in the regulation of T cell activation.

## RESULTS

### Human airway ECs inhibit T cell activation in a contact-independent manner

Previous studies have demonstrated that murine ECs are able to inhibit T cell proliferation [8–10]. Here, we first evaluated whether human airway ECs can inhibit T cell proliferation.

**Figure 1:**
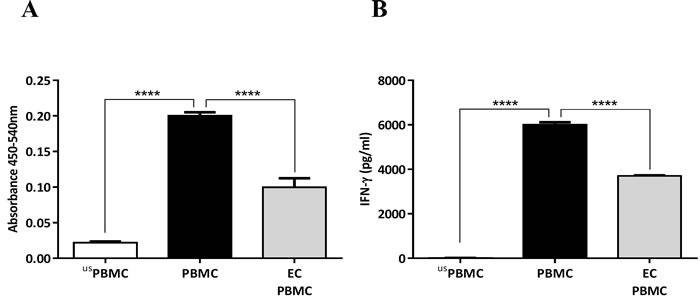
Inhibition of PBMC proliferation and IFN-γ production by human airway epithelial cells (ECs) **A.** Airway ECs (Calu-3) were co-cultured with PBMCs stimulated with 5 μg/ml of tuberculin purified protein derivate (PPD) without cell-cell contact (carried out in transwells^®^ with a 0.4-μm pore size). After six days, cell proliferation was assessed. **B.** IFN-γ concentration in culture supernatants. usPBMC= unstimulated PBMC, Data show mean ± SEM from three independent experiments. ****p < 0.0001.

ECs cultured on the apical side of a transwell membrane, were co-cultured with PBMCs (with no cell-cell contact) followed by stimulation with either PPD or anti-CD3 and CD28 Abs; and T cell proliferation was quantified after 3-6 days. The presence of ECs significantly suppressed PBMC proliferation (Figure 1A) and IFN-γ production (Figure 1B). We also performed the same experiments using a co-culture of DCs and purified T cells, which showed a similar level of suppression of T cell proliferation in the presence of ECs (data not shown).

### Stimulation of ECs with TLR3 and TLR4 ligands ameliorates their ability in suppressing T cell activation

**Figure 2:**
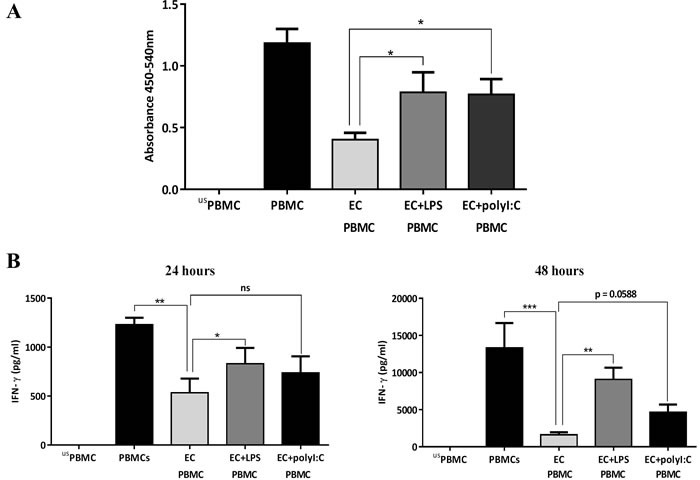
Influence of TLR-activated airway epithelial cells (ECs) on PBMC proliferation **A.** Human airway ECs (Calu-3) were co-cultured with PBMCs stimulated with 2 μg/ml of anti-CD3 and anti-CD28 in the presence of 10 μg/ml of LPS or polyribocytidylic acid (PolyI:C). After three days, proliferation was assessed. **B.** IFN-γ production in culture supernatants. Data show mean ± SEM from three independent experiments. us=unstimulated*p < 0.05, **p < 0.01, ***p < 0.001, ns, not significant.

We next investigated whether TLR agonists, simulating bacterial or viral infections, modulated the observed inhibitory effect of airway ECs on T cell activation. Therefore, we exposed airway ECs to TLR4 or TLR3 ligands (LPS and polyI:C, respectively) followed by their co-culture with anti-CD3/CD28-stimulated PBMCs. After three days, the cells were collected for a BrdU proliferation assay and the supernatant was collected for cytokine analysis. LPS and polyI:C treatment of ECs led to a clear reduction in their ability to suppress T cell proliferation compared with the control (Figure 2A). The degree of this effect correlated with the level of IFN-γ production, which showed a significant increase in response to LPS stimulation after 24 h and 48 h, and was also slightly enhanced in response to polyI:C, although this change was not statistically significant (Figure 2B). These findings suggest that the ability of ECs to regulate T cell activation could be significantly compromised following bacterial or viral infections.

### TLR4 stimulation down-regulates IDO in human airway ECs without compromising cell viability

**Figure 3:**
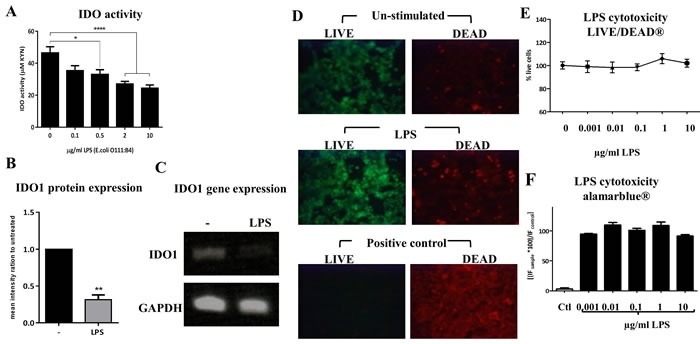
Down-regulation of indoleamine 2,3-dioxygenase (IDO) in response to LPS stimulation in human airway epithelial cells (ECs) **A.** Human airway ECs were incubated with increasing concentration of *E. coli* LPS for 24 h and IDO activity was assessed (*n* = 5). **B.** and **C.** IDO1 protein and gene expression in airway ECs stimulated with 2 μg/ml of LPS for 24 h, respectively (*n* = 3). **D.** LIVE/DEAD staining of airway ECs stimulated with LPS (10 μg/ml) for 24 h. Cells treated with 4% formaldehyde were used as a control. **E.** The fluorescence quantification of the LIVE/DEAD staining expressed as percentage of live cells (*n* = 2). **F.** AlamarBlue fluorescence readouts of airway ECs stimulated with increasing concentrations of LPS for 24 h; cells treated with 4% formaldehyde were used as a control (*n* = 4). Ctl=control Data represent the mean values ± SEM. *p < 0.05, **p < 0.01, ****p < 0.0001.

**Figure 4:**
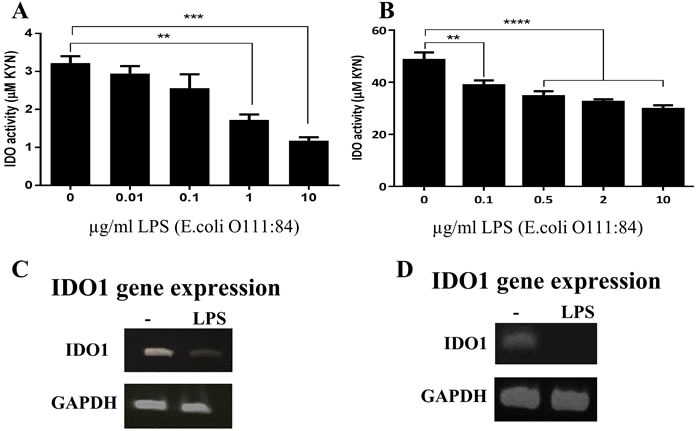
Down-regulation of indoleamine 2,3-dioxygenase (IDO) in response to LPS stimulation in human primary bronchial epithelial cells (HBECs) and cell lines **A.** and **B.** primary HBECs (*n* = 3) and HBEC line (BEAS-2B, *n* = 5) were incubated with increasing concentrations of *E. coli* LPS for 24 h, respectively, and IDO activity was assessed. (C and D) *IDO1* gene expression in response to stimulation with 2 μg/ml and 0.1 μg/ml of LPS in primary HBECs and BEAS-2B, respectively (*n* = 3). Data represent the mean values ± SEM. **p < 0.01, ***p < 0.001, ****p < 0.0001.

To test the hypothesis that the inhibition of T cell proliferation by ECs could be mediated by IDO, we first investigated the levels of IDO expression in ECs before and after stimulation with the TLR4 ligand LPS. Initially, we used the Calu-3 cell line as a model for human ECs. These cells were incubated with increasing concentration of LPS (from *Escherichia coli*) for 24 h, and IDO activity and expression were assessed. Significant down-regulation in IDO activity was observed (Figure 3A) in response to LPS stimulation. This reduction in IDO activity was accompanied by suppression in IDO1 protein (Figure 3B) and gene (Figure 3C) expression. LPS from *Salmonella minnesota* had the same effect on IDO activity (data not shown). To determine whether cell death contributed to the observed reduction in IDO activity, we measured the viability of ECs after LPS stimulation using AlamarBlue and LIVE/DEAD staining. The data showed comparable EC viability before and after LPS stimulation (Figure 3D), indicating that the substantial reduction of IDO expression was not associated with cellular death. The same pattern of a reduction in IDO activity in response to LPS was also observed in human primary ECs as well as in a non-cancerous epithelial cell line (BEAS-2B), highlighting the conserved nature of IDO regulation by LPS (Figure 4).

### Diverse TLR ligands and allergen extracts decrease IDO activity and expression in human airway ECs

**Figure 5:**
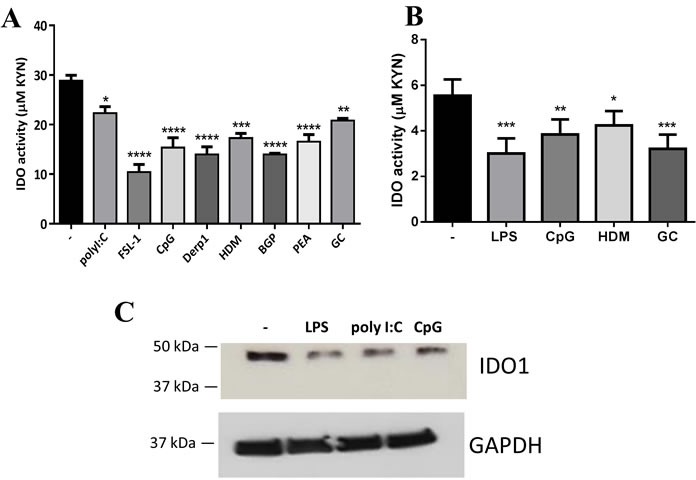
Diverse TLR ligands and allergen extracts decreased indoleamine 2,3-dioxygenase (IDO) expression in human airway epithelial cells (ECs) **A.** Airway ECs (Calu-3) were incubated with 10 μg/ml of different TLR ligands and allergen extracts for 24 h (n ≥ 3). **B.** Primary human bronchial ECs were incubated with 2 μg/ml LPS, 10 μg/ml CpG, and 10 μg/ml allergen extracts (*n* = 3). **C.** Calu-3 ECs were treated with 10 μg/ml LPS, 10 g/ml polyribocytidylic acid (PolyI:C), or 10 g/ml CpG for 24 h, and IDO1 protein levels were assessed in cell lysates by western blot analysis; GAPDH was used as a control (*n* = 3). Observed band sizes were 45 kDa for IDO1 and 36 kDa for GAPDH, as predicted. HDM, house dust mite; BGP, Bermuda grass pollen; PEA, peanut; GC, German cockroach. Data represent the mean values ± SEM. *p < 0.05, **p < 0.01, ***p < 0.001, ****p < 0.0001.

To verify whether IDO downregulation is only observed in response to LPS, we next sought to determine the impact of other TLR ligands as well as different allergen extracts on IDO activity in human airway ECs. ECs were incubated for 24 h with 10 μg/ml of various TLR ligands or allergen extracts. As shown in Figure 5, TLR3 (polyI:C), TLR2/6 (FSL-1), and TLR9 (CpG) engagement markedly inhibited IDO activity. However, TLR7 and TLR1/2 ligation with imiquimod and a synthetic triacylated lipopeptide (Pam3CSK4) respectively did not affect IDO activity levels in ECs (data not shown). Furthermore, we found significant inhibition of IDO activity in response to different allergen extracts, including Bermuda grass pollen (BGP), peanut (PEA), German cockroach (GC), house dust mite (HDM) and its purified allergen Der p 1 (Figure 5A). The down-regulation of IDO activity was also observed in primary ECs in response to LPS, CpG, and different allergen extracts (Figure 5B). To examine whether the suppression in IDO activity was reflected in its expression at the protein level, we also quantified IDO1 protein expression levels in ECs before and after TLR ligation using western blotting. These experiments showed a marked reduction in IDO1 protein expression in TLR-stimulated ECs compared to unstimulated cells (Figure 5C).

### IDO contributes to the airway ECs-mediated suppression of T cell activation

**Figure 6:**
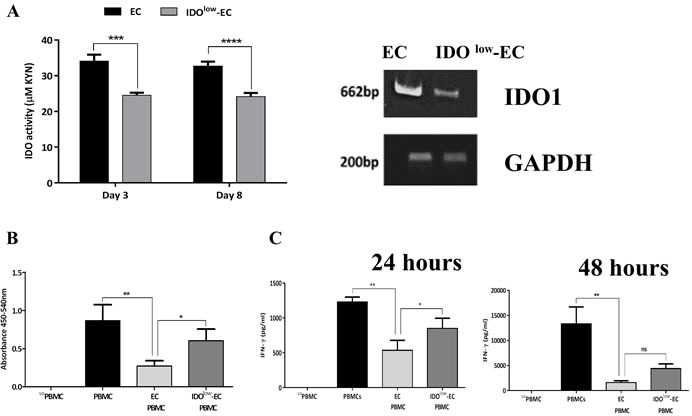
Role of indoleamine 2,3-dioxygenase (IDO)-expressing airway epithelial cells (ECs) on inhibition of PBMCs activation **A.** Human airway ECs were transfected for 6 h with 50 nM of IDO1 small interfering RNA (siRNA) using a non-targeting siRNA as a negative control. The efficiency of IDO downregulation after transfection was assessed by evaluation of IDO activity and *IDO1* mRNA expression (*n* = 3). **B.** Control ECs (i.e., IDO^high^) and IDO^low^-ECs were co-cultured with anti-CD3-activated PBMCs. After three days, proliferation was assessed (*n* = 4). **C.** IFN-γ levels in the culture supernatants were quantified after 24 h and 48 h (*n* = 3). Data represent the mean values ± SEM. *p < 0.05, **p < 0.01, ***p < 0.001, ****p < 0.0001; ns, not significant.

Previous findings have suggested a role for EC-produced TGF-β in modulating T cell proliferation [9]. However, the exact mechanism by which ECs inhibit T cell proliferation remains unclear. We therefore examined the potential role of IDO in mediating the inhibitory effect of ECs on T cells. First, we attempted to block IDO activity in ECs/PBMCs co-cultures using 1-methyl tryptophan, an IDO-inhibitor. However, given the direct effect of 1-methyl tryptophan on PBMCs leading to suppression of their proliferation (data not shown), we decided to directly target IDO activity in ECs. Thus, we generated IDO^low^-ECs by knocking down the *IDO1* gene in ECs using small interfering RNA (siRNA), so that only IDO activity was suppressed in ECs. As shown in Figure 6A, there was a significant decrease in IDO activity and *IDO1* mRNA levels after gene silencing (i.e., IDO^low^) compared with the control cells that expressed high levels of IDO. Subsequently, we co-cultured the IDO^low^-ECs with anti-CD3-activated PBMCs, and proliferation was assessed three days later. Figure 6B shows that, as expected, IDO-sufficient ECs significantly inhibited T cell proliferation, and this effect was significantly reduced after silencing IDO. We also investigated cytokine production after 24 h and 48 h. As expected, we observed a significant reduction in IFN-γ production in the presence of ECs and a partial reconstitution in IFN-γ levels in the presence of IDO^low^-ECs compared with the control cells (Figure 6C). Collectively, these results demonstrated the important role of IDO produced by ECs for the inhibition of T cell activation.

## DISCUSSION

ECs are one of the main cellular components of the innate immune system, which provide primary protection against airborne pathogens and allergens. Previous studies have shown that ECs can directly regulate conventional and unconventional T cell responses [8, 9, 32, 33]; however, the molecular basis of such regulation has remained elusive. We hypothesized that airway ECs modulate human T cell responses *via* expression of IDO. Our data showed that airway ECs could suppress antigen-specific and non-specific T cell activation (i.e., T cell proliferation and IFN-γ production). Furthermore, the levels of T cell activation were partially recovered when ECs were exposed to different TLR agonists as mimics for bacterial and viral infections. Such reduction in the immune-suppressive effect of ECs after stimulation with bacterial and viral products could be crucial for facilitating the development of appropriate inflammatory responses for containing the infection.

IDO is the TRP-depleting enzyme in the KYN pathway and has been extensively associated with modulating immune responses. DCs are one of the main producers of IDO-derived metabolites in the immune system [17, 18]; however, over the last decade ECs have also emerged as key players in modulating immune responses in the airways. Upon stimulation with allergens, airway ECs are able to produce a wide range of innate mediators that can activate other immune cells, including DCs, basophils and innate lymphoid cells. In addition, ECs express functional IDO after stimulation with pro-inflammatory cytokines such as IFN-γ and TNF-α [23, 24, 26–29].

It is widely recognised that IDO is constitutively expressed by many tumors as well as by infiltrating leucocytes present within the tumor microenvironment [34–36]. IDO is enzymatically active in human cancerous cell lines and creates an immunosuppressive microenvironment *via* TRP depletion and by formation of immunosuppressive IDO metabolites [37, 38]. In tumor state, high IDO expression and activity provides systemic tolerance *via* inhibition of the effector T cell infiltration [34, 39] and induction of Treg formation [40]. In addition, IDO could promote tumor cell survival and motility *via* production of endogeneuos KYN that drive the activation of aryl hydrocarbon receptor in cancer cells [16]. It has been reported that induction of IDO *via* IFN-γ provides anti-tumor effector mechanism in diverse human cancer cell lines including epithelial cell lines through the inhibition of their proliferation *via* TRP starvation [41]. Taken together, it is evident that elevated level of IDO in tumor environment could provide local and systemic immune suppression to facilitate immune escape by cancer cells [35]. Here in this study, we show for the first time that human airway ECs constitutively express IDO under resting conditions and critically there is significant down-regulation of IDO activity in both primary ECs and the cell lines in response to a wide range of TLR ligands and allergen extracts. Although the basal level of KYN production by primary human bronchial epithelial cells was lower (~3-6 μM) than that in BEAS-2B or Calu-3 cell lines (~20-40 μM) both cell types were similarly susceptible to stimulation with TLR ligands and allergens. Previous studies have suggested nitric oxide (NO) can inhibit IDO activity and expression [26, 42] most likely through post-translational regulation leading to proteasomal degradation of IDO rather than transcriptional regulation [26]. Down-regulation in IDO protein levels has also been observed in DCs through IL-6 mediated proteasomal degradation [43, 44]. Since our data show significant transcriptional changes in IDO levels in ECs, hence it is reasonable to assume that the observed transcriptional changes in IDO in our experiments are unlikely to be due to changes in IL-6 or NO levels, and rather involve activation of intracellular signaling pathways that ultimately modulate IDO gene expression. Indeed this is in line with our observation showing no changes in IL-6 levels in all our experimental conditions (data not shown).

Interestingly, the down-regulation of IDO in response to these stimuli in ECs is in contrast to the responses previously observed in DCs and macrophages [21, 45, 46], in which IDO activity is generally up-regulated. This finding thus highlights the complexity of IDO regulation and fundamental differences in its activity between different cell types. It is reasonable to suggest that the constitutive expression of IDO by ECs under steady-state conditions is associated with their ability to suppress unwanted immune reactions and to maintain immune homeostasis in the airways. In the context of cancerous cell lines, the high level of IDO activity contributes to the immunosuppressive tumor environment. However, after encountering pathogens (i.e., TLR engagement) and/or allergens (i.e., TLR or C-type lectin engagement), this regulatory mechanism could be disrupted through down-regulation of IDO activity, which in turn could lead to increased T cell activity and subsequent immune responses to combat the infection.

Previous studies have shown the importance of IDO from ECs during viral, bacterial, and parasite infections [26, 28, 31, 47–53]. Moreover, increased susceptibility to infection has been associated with the development of asthma-related diseases [54]. Accordingly, it would be interesting to explore the role of IDO and specific IDO-derived metabolites from airway ECs in modulating other immune and non-immune cells in the context of respiratory infection.

We further evaluated the direct role of IDO in ECs in modulating T cell activation by generating IDO^low^-ECs using gene silencing. We found that knocking down IDO activity in ECs significantly reduced their ability to suppress T cell proliferation and IFN-γ production, suggesting an important role for IDO in this process. This effect is likely to be driven by a reduction in the level of IDO metabolites such as KYN and 3-HAA which are thought to drive most of the IDO-immune-suppressive effects [20, 55–60] however the exact mechanism is yet to be elucidated. It is also important to emphasize that the effects of IDO metabolites produced by ECs may not be limited to T cells, and might also modulate the function of other immune cells (e.g. basophils, and mast cells), broadening their overall immune modulatory effect. Future work should explore such possibilities.

In conclusion, our results underline the important role of airway ECs in controlling immune responses through direct modulation of T cell function *via* production of IDO. Given the pivotal role of IDO-derived metabolites from DCs in modulating immune responses in the airways [21, 61–64] and the evidence pointing to a key role of EC-derived cytokines in modulating DC behavior [54], it would be interesting to study the effect of the IDO pathway on the cross-talk between DCs and ECs in the context of inflammatory conditions affecting the airways. This would help to better understand several airway-related pathologies such as allergic asthma and respiratory infections, leading to the development of improved therapeutic strategies to combat deleterious inflammatory responses in the airways.

## MATERIALS AND METHODS

### Lung epithelial cells

Calu-3 cells (ATCC^®^ HTB55™, a human lung epithelial adenocarcinoma cell line) were maintained in DMEM-Ham's Nutrient Mixture F-12 (DMEM-F12; Sigma-Aldrich, St. Louis, MO, USA) containing 10% v/v heat-inactivated FBS, 100 U/ml penicillin-100 μg/ml streptomycin (1% v/v pen/strep), 2 mM l-glutamine, and 1% v/v non-essential amino acid solution (Sigma-Aldrich, St. Louis, MO, USA). BEAS-2B cells (ATTC^®^ CRL-9609^TM^, a human bronchial epithelial cell line) were maintained in DMEM (Sigma-Aldrich, St. Louis, MO, USA) with 10% v/v heat-inactivated FBS, 100 U/ml penicillin-100 μg/ml streptomycin, and 2 mM l-glutamine. Primary human bronchial ECs were separated from the bronchial brushings (from healthy volunteers attending Nottingham University Hospital Respiratory Clinic after ethics committee approval and obtaining informed written consent) and were maintained in basal Bronchial Epithelial Growth Media supplemented with BEGM™ SingleQuots™ Kit (Clonetics™ Airway Epithelial Cell Systems, Lonza).

### PBMCs separation

PBMCs were isolated from fresh blood samples of healthy volunteers (obtained after receiving informed consent and local Ethics Committee approval) by density-gradient centrifugation on Histopaque (Sigma-Aldrich, St. Louis, MO, USA). The cells were washed twice with PBS and 8 × 10^5^ cells/600 μl were co-cultured with Calu-3 cells in co-culture complete medium (1:1 ratio of DMEM-F12:RPMI-1640; Sigma-Aldrich, St. Louis, MO, USA).

### IDO enzymatic activity assay

Calu-3 cells (2 × 10^5^ cells/ml) were seeded in a 24-well plate supplemented with 100 μM l-TRP (Sigma-Aldrich, St. Louis, MO, USA), and grown to confluence for 24 h. Cells were stimulated with various TLR agonists and allergen extracts, including LPS and polyribocytidylic acid (polyI:C) (both from Sigma-Aldrich, St. Louis, MO, USA), synthetic lipoprotein of *Mycoplasma salivarium* (FSL-1), CpG-ODN2216 (5′-ggGGGACGA:TCGTCgggggg-3′) (both from Invivogen), house dust mite (Greer laboratories) extract and its purified allergen (Der p 1, Indoor Biotechnologies), Bermuda grass pollen, peanut, and German cockroach extracts (all from Greer laboratories). IDO activity was measured by quantification of kynurenines in the culture supernatant using a colorimetric assay as we have described previously [64].

### Viability assay

Cell viability was quantified using the LIVE/DEAD viability/cytotoxicity kit and AlamarBlue assay (both from Invitrogen) as described previously [65, 66].

### mRNA isolation, cDNA synthesis and PCR

mRNA isolation and cDNA synthesis were performed using the μMACS one-step cDNA kit (Miltenyi Biotech) following the manufacturer instructions. PCR was carried out in a TC-312 PCR Thermocycler (Bibby Scientific Ltd.) using the Phusion Flash High-Fidelity PCR Master Mix (Thermo Scientific). *IDO1* specific mRNA expression was quantified relative to glyceraldehyde 3-phosphate dehydrogenase (*GAPDH*) expression using the following primers (Eurofins): *GAPDH* Forward (5′-GAGTCAACGGATTTGGTCGT-3′), *GAPDH* Reverse (5′-GACAAGCTTCCCGTTCTCAG-3′), *IDO1* Forward (5′-ACAGACCACAAGTCACAGCG-3′) and *IDO1* Reverse (5′-AACTGAGCAGCATGTCCTCC-3′). PCR products were analyzed on a 2% agarose gel electrophoresis system (Invitrogen, UK), visualized under UV light (Alpha Innotech Corporation), and the molecular weights of the bands were estimated with a standard 100-bp Directload DNA ladder (Sigma-Aldrich, St. Louis, MO, USA).

### Western blotting

Cells were washed with ice-cold PBS and then lysed using ice-cold radio-immunoprecipitation assay lysis buffer with protease and phosphatase inhibitor (Sigma-Aldrich, St. Louis, MO, USA) on a shaker for 30 min at 4°C. Subsequently, cell lysates were centrifuged at 13,000 rpm for 30 min at 4°C. Supernatants were collected and stored at −20°C. Protein concentration was determined using the DC Protein Assay (Biorad) following the manufacturer's protocol. The cell lysate was electrophoresed under reducing and denaturating conditions on a pre-cast 4-15% SDS-polyacrylamide gel (Biorad), and proteins were transferred to a nitrocellulose membrane (GE Healthcare). After a 2 h incubation in blocking buffer (5% non-fat milk in Tris-glycine buffer and 0.1% Tween 20), membranes were incubated with a primary rabbit monoclonal antibody [EPR1230Y] to IDO (Abcam, Cambridge, UK) overnight. After washing, the secondary peroxidase-conjugated antibody (goat anti-rabbit HRP) was added for 1 h. IDO was visualized using Amersham ECL prime Western Blotting Detection Reagents (GE Healthcare). GAPDH was used as a loading control with the rabbit monoclonal antibody (Abcam, Cambridge, UK).

### Gene silencing

Calu-3 cells were transfected with *IDO1*-specific siRNA (GE Healthcare, UK) according to the manufacturer instructions. In brief, the cells were transfected on day one with 50 nM of *IDO1* siRNA using a non-targeting siRNA as a negative control. The cells were then incubated for 6 h in complete medium without penicillin/streptomycin in a low-attachment tissue culture plate (Corning Life Sciences, Tewksbury, MA, USA) and then left for three days. The efficiency of IDO down-regulation was assessed by an IDO activity quantitative assay as well as with conventional PCR for determination of *IDO1* mRNA expression.

### Co-culture conditions

Control or IDO^low^-ECs (6 × 10^5^ cells/ml) were cultured in Transwells^®^ with a 0.4 μm pore size (Scientific Laboratory Supplies Ltd.). After reaching confluence, PBMCs (8 × 10^5^ cells/600 μl) were cultured in the bottom chamber in the presence of immobilized anti-CD3 antibody (2 μg/ml) (Sigma-Aldrich, St. Louis, MO, USA) or 5 μg/ml of tuberculin purified protein derivate (Statens Serum Institute). In some experiments, the ECs were stimulated with TLR ligands. In addition, under certain conditions, IDO metabolites (KYN, 3-HAA and its vehicle) and HCl (all from Sigma-Aldrich, St. Louis, MO, USA) were added to the PBMCs. The cells were collected after 3-6 days for a BrdU proliferation assay and the supernatants were stored for cytokine analysis.

### Proliferation assay

Cell proliferation was determined with the BrdU-ELISA assay (Calbiochem, USA) according to the manufacturer instructions.

### Cytokine measurements

Cytokine concentrations in the culture supernatants incubated for 24 h and 48 h were assessed using the DuoSet ELISA system for human IFN-γ (R&D Systems, UK) following the manufacturer instructions.

### Statistical analysis

Data are expressed as mean values ± SEM. Statistical differences were determined using the Student *t*-test to compare two groups and using one-way ANOVA to compare three groups or more. Statistical significance was associated with *p* values < 0.05.

## References

[1] Willart M, Hammad H (2011). Lung dendritic cell-epithelial cell crosstalk in Th2 responses to allergens. Current opinion in immunology.

[2] Hammad H, Lambrecht BN (2011). Dendritic cells and airway epithelial cells at the interface between innate and adaptive immune responses. Allergy.

[3] Reibman J, Hsu Y, Chen LC, Bleck B, Gordon T (2003). Airway epithelial cells release MIP-3alpha/CCL20 in response to cytokines and ambient particulate matter. American journal of respiratory cell and molecular biology.

[4] Pichavant M, Charbonnier AS, Taront S, Brichet A, Wallaert B, Pestel J, Tonnel AB, Gosset P (2005). Asthmatic bronchial epithelium activated by the proteolytic allergen Der p 1 increases selective dendritic cell recruitment. The Journal of allergy and clinical immunology.

[5] Stumbles PA, Strickland DH, Pimm CL, Proksch SF, Marsh AM, McWilliam AS, Bosco A, Tobagus I, Thomas JA, Napoli S, Proudfoot AE, Wells TN, Holt PG (2001). Regulation of dendritic cell recruitment into resting and inflamed airway epithelium: use of alternative chemokine receptors as a function of inducing stimulus. Journal of immunology.

[6] Soumelis V, Reche PA, Kanzler H, Yuan W, Edward G, Homey B, Gilliet M, Ho S, Antonenko S, Lauerma A, Smith K, Gorman D, Zurawski S (2002). Human epithelial cells trigger dendritic cell mediated allergic inflammation by producing TSLP. Nature immunology.

[7] Ito T, Wang YH, Duramad O, Hori T, Delespesse GJ, Watanabe N, Qin FX, Yao Z, Cao W, Liu YJ (2005). TSLP-activated dendritic cells induce an inflammatory T helper type 2 cell response through OX40 ligand. The Journal of experimental medicine.

[8] Cruickshank SM, McVay LD, Baumgart DC, Felsburg PJ, Carding SR (2004). Colonic epithelial cell mediated suppression of CD4 T cell activation. Gut.

[9] Wang H, Su Z, Schwarze J (2009). Healthy but not RSV-infected lung epithelial cells profoundly inhibit T cell activation. Thorax.

[10] Albrecht M, Arnhold M, Lingner S, Mahapatra S, Bruder D, Hansen G, Dittrich AM (2012). IL-4 Attenuates Pulmonary Epithelial Cell-Mediated Suppression of T Cell Priming. PLoS One.

[11] Papazian D, Wagtmann VR, Hansen S, Wurtzen PA (2015). Direct contact between dendritic cells and bronchial epithelial cells inhibits T cell recall responses towards mite and pollen allergen extracts in vitro. Clinical and experimental immunology.

[12] Schmidt SK, Muller A, Heseler K, Woite C, Spekker K, MacKenzie CR, Daubener W (2009). Antimicrobial and immunoregulatory properties of human tryptophan 2 3-dioxygenase. European journal of immunology.

[13] Moffett JR, Namboodiri MA (2003). Tryptophan and the immune response. Immunology and cell biology.

[14] Platten M, von Knebel Doeberitz N, Oezen I, Wick W, Ochs K (2014). Cancer Immunotherapy by Targeting IDO1/TDO and Their Downstream Effectors. Frontiers in immunology.

[15] Opitz CA, Litzenburger UM, Opitz U, Sahm F, Ochs K, Lutz C, Wick W, Platten M (2011). The indoleamine-2 3-dioxygenase (IDO) inhibitor 1-methyl-D-tryptophan upregulates IDO1 in human cancer cells. PloS one.

[16] Platten M, Wick W, Van den Eynde BJ (2012). Tryptophan catabolism in cancer: beyond IDO and tryptophan depletion. Cancer research.

[17] Munn DH, Mellor AL (2012). Indoleamine 2 3 dioxygenase and metabolic control of immune responses. Trends in immunology.

[18] Orabona C, Pallotta MT, Grohmann U (2012). Different partners opposite outcomes: A new perspective of IDO's immunobiology. Molecular medicine.

[19] Munn DH, Sharma MD, Baban B, Harding HP, Zhang Y, Ron D, Mellor AL (2005). GCN2 kinase in T cells mediates proliferative arrest and anergy induction in response to indoleamine 2 3-dioxygenase. Immunity.

[20] Mezrich JD, Fechner JH, Zhang X, Johnson BP, Burlingham WJ, Bradfield CA (2010). An interaction between kynurenine and the aryl hydrocarbon receptor can generate regulatory T cells. Journal of immunology.

[21] Salazar F, Hall L, Negm OH, Awuah D, Tighe PJ, Shakib F, Ghaemmaghami AM (2016). The mannose receptor negatively modulates the Toll-like receptor 4-aryl hydrocarbon receptor-indoleamine 2 3-dioxygenase axis in dendritic cells affecting T helper cell polarization. The Journal of allergy and clinical immunology.

[22] Allegri G, Ragazzi E, Bertazzo A, Costa CV (2003). Enzyme activities along the kynurenine pathway in mice. Adv Exp Med Biol.

[23] Babcock TA, Carlin JM (2000). Transcriptional activation of indoleamine dioxygenase by interleukin 1 and tumor necrosis factor alpha in interferon-treated epithelial cells. Cytokine.

[24] Robinson CM, Shirey KA, Carlin JM (2003). Synergistic transcriptional activation of indoleamine dioxygenase by IFN-gamma and tumor necrosis factor-alpha. Journal of interferon & cytokine research.

[25] Du MX, Sotero-Esteva WD, Taylor MW (2000). Analysis of transcription factors regulating induction of indoleamine 2 3-dioxygenase by IFN-gamma. Journal of interferon & cytokine research.

[26] Hucke C, MacKenzie CR, Adjogble KD, Takikawa O, Daubener W (2004). Nitric oxide-mediated regulation of gamma interferon-induced bacteriostasis: inhibition and degradation of human indoleamine 2 3-dioxygenase. Infection and immunity.

[27] Bell LV, Else KJ (2011). Regulation of colonic epithelial cell turnover by IDO contributes to the innate susceptibility of SCID mice to Trichuris muris infection. Parasite immunology.

[28] Bodaghi B, Goureau O, Zipeto D, Laurent L, Virelizier JL, Michelson S (1999). Role of IFN-gamma-induced indoleamine 2 3 dioxygenase and inducible nitric oxide synthase in the replication of human cytomegalovirus in retinal pigment epithelial cells. Journal of immunology.

[29] Fougeray S, Mami I, Bertho G, Beaune P, Thervet E, Pallet N (2012). Tryptophan depletion and the kinase GCN2 mediate IFN-gamma-induced autophagy. Journal of immunology.

[30] Takikawa O, Yoshida R, Kido R, Hayaishi O (1986). Tryptophan degradation in mice initiated by indoleamine 2 3-dioxygenase. The Journal of biological chemistry.

[31] Jiang N, Zhao G, Lin J, Hu L, Che C, Li C, Wang Q, Xu Q, Peng X (2015). Indoleamine 2 3-Dioxygenase Is Involved in the Inflammation Response of Corneal Epithelial Cells to Aspergillus fumigatus Infections. PLoS One.

[32] Strid J, Sobolev O, Zafirova B, Polic B, Hayday A (2011). The intraepithelial T cell response to NKG2D-ligands links lymphoid stress surveillance to atopy. Science.

[33] Gereke M, Jung S, Buer J, Bruder D (2009). Alveolar type II epithelial cells present antigen to CD4(+) T cells and induce Foxp3(+) regulatory T cells. American journal of respiratory and critical care medicine.

[34] Litzenburger UM, Opitz CA, Sahm F, Rauschenbach KJ, Trump S, Winter M, Ott M, Ochs K, Lutz C, Liu X, Anastasov N, Lehmann I, Hofer T, von Deimling A, Wick W, Platten M (2014). Constitutive IDO expression in human cancer is sustained by an autocrine signaling loop involving IL-6 STAT3 and the AHR. Oncotarget.

[35] Yeung AW, Terentis AC, King NJ, Thomas SR (2015). Role of indoleamine 2 3-dioxygenase in health and disease. Clinical science.

[36] Munn DH, Sharma MD, Hou D, Baban B, Lee JR, Antonia SJ, Messina JL, Chandler P, Koni PA, Mellor AL (2004). Expression of indoleamine 2 3-dioxygenase by plasmacytoid dendritic cells in tumor-draining lymph nodes. The Journal of clinical investigation.

[37] Theate I, van Baren N, Pilotte L, Moulin P, Larrieu P, Renauld JC, Herve C, Gutierrez-Roelens I, Marbaix E, Sempoux C, Van den Eynde BJ (2015). Extensive profiling of the expression of the indoleamine 2 3-dioxygenase 1 protein in normal and tumoral human tissues. Cancer immunology research.

[38] Munn DH, Mellor AL (2007). Indoleamine 2 3-dioxygenase and tumor-induced tolerance. The Journal of clinical investigation.

[39] Brandacher G, Perathoner A, Ladurner R, Schneeberger S, Obrist P, Winkler C, Werner ER, Werner-Felmayer G, Weiss HG, Gobel G, Margreiter R, Konigsrainer A, Fuchs D, Amberger A (2006). Prognostic value of indoleamine 2 3-dioxygenase expression in colorectal cancer: effect on tumor-infiltrating T cells. Clinical cancer research.

[40] Brody JR, Costantino CL, Berger AC, Sato T, Lisanti MP, Yeo CJ, Emmons RV, Witkiewicz AK (2009). Expression of indoleamine 2,3-dioxygenase in metastatic malignant melanoma recruits regulatory T cells to avoid immune detection and affects survival. Cell Cycle.

[41] Aune TM, Pogue SL (1989). Inhibition of tumor cell growth by interferon-gamma is mediated by two distinct mechanisms dependent upon oxygen tension: induction of tryptophan degradation and depletion of intracellular nicotinamide adenine dinucleotide. The Journal of clinical investigation.

[42] Hara T, Ogasawara N, Akimoto H, Takikawa O, Hiramatsu R, Kawabe T, Isobe K, Nagase F (2008). High-affinity uptake of kynurenine and nitric oxide-mediated inhibition of indoleamine 2,3-dioxygenase in bone marrow-derived myeloid dendritic cells. Immunology letters.

[43] Grohmann U, Fallarino F, Bianchi R, Belladonna ML, Vacca C, Orabona C, Uyttenhove C, Fioretti MC, Puccetti P (2001). IL-6 inhibits the tolerogenic function of CD8 alpha+ dendritic cells expressing indoleamine 2,3-dioxygenase. Journal of immunology.

[44] Fallarino F, Grohmann U, Puccetti P (2012). Indoleamine 2,3-dioxygenase: from catalyst to signaling function. European journal of immunology.

[45] Munn DH, Mellor AL (2013). Indoleamine 2,3 dioxygenase and metabolic control of immune responses. Trends in immunology.

[46] Munn DH, Shafizadeh E, Attwood JT, Bondarev I, Pashine A, Mellor AL (1999). Inhibition of T cell proliferation by macrophage tryptophan catabolism. The Journal of experimental medicine.

[47] Schmidt SV, Schultze JL (2014). New Insights into IDO Biology in Bacterial and Viral Infections. Frontiers in immunology.

[48] Huang L, Li L, Klonowski KD, Tompkins SM, Tripp RA, Mellor AL (2013). Induction and role of indoleamine 2,3 dioxygenase in mouse models of influenza a virus infection. PLoS One.

[49] Nagineni CN, Pardhasaradhi K, Martins MC, Detrick B, Hooks JJ (1996). Mechanisms of interferon-induced inhibition of Toxoplasma gondii replication in human retinal pigment epithelial cells. Infection and immunity.

[50] Adams O, Besken K, Oberdorfer C, MacKenzie CR, Russing D, Daubener W (2004). Inhibition of human herpes simplex virus type 2 by interferon gamma and tumor necrosis factor alpha is mediated by indoleamine 2,3-dioxygenase. Microbes and infection.

[51] Obojes K, Andres O, Kim KS, Daubener W, Schneider-Schaulies J (2005). Indoleamine 2,3-dioxygenase mediates cell type-specific anti-measles virus activity of gamma interferon. Journal of virology.

[52] Loughman JA, Hunstad DA (2012). Induction of indoleamine 2,3-dioxygenase by uropathogenic bacteria attenuates innate responses to epithelial infection. The Journal of infectious diseases.

[53] Desvignes L, Ernst JD (2009). Interferon-gamma-responsive nonhematopoietic cells regulate the immune response to Mycobacterium tuberculosis. Immunity.

[54] Lambrecht BN, Hammad H (2015). The immunology of asthma. Nature immunology.

[55] Fallarino F, Grohmann U, Vacca C, Bianchi R, Orabona C, Spreca A, Fioretti MC, Puccetti P (2002). T cell apoptosis by tryptophan catabolism. Cell death and differentiation.

[56] Hayashi T, Mo JH, Gong X, Rossetto C, Jang A, Beck L, Elliott GI, Kufareva I, Abagyan R, Broide DH, Lee J, Raz E (2007). 3-Hydroxyanthranilic acid inhibits PDK1 activation and suppresses experimental asthma by inducing T cell apoptosis. Proceedings of the National Academy of Sciences of the United States of America.

[57] Mbongue JC, Nicholas DA, Torrez TW, Kim NS, Firek AF, Langridge WH (2015). The Role of Indoleamine 2, 3-Dioxygenase in Immune Suppression and Autoimmunity. Vaccines.

[58] Bauer TM, Jiga LP, Chuang JJ, Randazzo M, Opelz G, Terness P (2005). Studying the immunosuppressive role of indoleamine 2,3-dioxygenase: tryptophan metabolites suppress rat allogeneic T-cell responses in vitro and in vivo. Transplant international.

[59] Terness P, Bauer TM, Rose L, Dufter C, Watzlik A, Simon H, Opelz G (2002). Inhibition of allogeneic T cell proliferation by indoleamine 2,3-dioxygenase-expressing dendritic cells: mediation of suppression by tryptophan metabolites. The Journal of experimental medicine.

[60] Nguyen NT, Kimura A, Nakahama T, Chinen I, Masuda K, Nohara K, Fujii-Kuriyama Y, Kishimoto T (2010). Aryl hydrocarbon receptor negatively regulates dendritic cell immunogenicity via a kynurenine-dependent mechanism. Proceedings of the National Academy of Sciences of the United States of America.

[61] Grohmann U, Volpi C, Fallarino F, Bozza S, Bianchi R, Vacca C, Orabona C, Belladonna ML, Ayroldi E, Nocentini G, Boon L, Bistoni F, Fioretti MC, Romani L, Riccardi C, Puccetti P (2007). Reverse signaling through GITR ligand enables dexamethasone to activate IDO in allergy. Nature medicine.

[62] Taher YA, Piavaux BJ, Gras R, van Esch BC, Hofman GA, Bloksma N, Henricks PA, van Oosterhout AJ (2008). Indoleamine 2,3-dioxygenase-dependent tryptophan metabolites contribute to tolerance induction during allergen immunotherapy in a mouse model. The Journal of allergy and clinical immunology.

[63] Maneechotesuwan K, Supawita S, Kasetsinsombat K, Wongkajornsilp A, Barnes PJ (2008). Sputum indoleamine-2, 3-dioxygenase activity is increased in asthmatic airways by using inhaled corticosteroids. The Journal of allergy and clinical immunology.

[64] Royer PJ, Emara M, Yang C, Al-Ghouleh A, Tighe P, Jones N, Sewell HF, Shakib F, Martinez-Pomares L, Ghaemmaghami AM (2010). The mannose receptor mediates the uptake of diverse native allergens by dendritic cells and determines allergen-induced T cell polarization through modulation of IDO activity. Journal of immunology.

[65] Htwe SS, Harrington H, Knox A, Rose F, Aylott J, Haycock JW, Ghaemmaghami AM (2015). Investigating NF-kappaB signaling in lung fibroblasts in 2D and 3D culture systems. Respiratory research.

[66] Zhao X, Lang Q, Yildirimer L, Lin ZY, Cui W, Annabi N, Ng KW, Dokmeci MR, Ghaemmaghami AM, Khademhosseini A (2016). Photocrosslinkable Gelatin Hydrogel for Epidermal Tissue Engineering. Advanced healthcare materials.

